# Is Oral Omega-3 Effective in Reducing Mucocutaneous Side Effects of Isotretinoin in Patients with Acne Vulgaris?

**DOI:** 10.1155/2018/6974045

**Published:** 2018-05-29

**Authors:** Mina Mirnezami, Hoda Rahimi

**Affiliations:** ^1^Department of Dermatology, Faculty of Medicine, Arak University of Medical Sciences, Arak, Iran; ^2^Skin Research Center, Shahid Beheshti University of Medical Sciences, Tehran, Iran

## Abstract

*Background. *Acne vulgaris is an inflammatory disease of pilosebaceous units which may cause permanent dyspigmentation and/or scars if not treated. Isotretinoin is recommended in the treatment of recalcitrant or severe acne, but it is associated with common adverse effects that frequently result in patients incompliance and discontinuation of the drug. The present study was designed to assess the efficacy of oral omega-3 in decreasing the adverse effects of isotretinoin.* Materials and Methods. *In this randomized double-blind clinical trial, a total of 118 patients with moderate or severe acne were randomly divided into two (case and control) groups. The control group was treated with isotretinoin 0.5 mg/kg, and the case group was treated with the same dose of isotretinoin combined with oral omega-3 (1 g/day). The treatment was lasted for 16 weeks and mucocutaneous side effects of isotretinoin were recorded and compared between the two groups in weeks 4, 8, 12, and 16.* Results. *Cheilitis (at weeks 4, 8, and 12), xerosis, dryness of nose at all weeks, and dryness of eyes (at week 4) were less frequent in the group that received isotretinoin combined with oral omega-3 compared to the group that received isotretinoin alone.* Conclusion.* Administration of oral omega-3 in acne patients who are receiving isotretinoin decreases the mucocutaneous side effects of isotretinoin.* This trial is registered with*  IRCT201306238241N2.

## 1. Introduction

Acne vulgaris is a common disease of the pilosebaceous units that affects both sexes during puberty. Although, severity and duration of acne vary in different people [1], its severe types are more common in males and its long-term forms are more common in women. Some studies have reported prevalence rate of 85% among people aged 12 to 25 [2, 3]. Mild acne resolves without scars, but the severe types may cause permanent scars [2–4]. Lesions are more common and severe in areas of the skin rich in sebaceous glands [5]. During puberty, following sebum production,* Propionibacterium* acne proliferates in the skin and some alterations occur in the follicular epithelial membrane, resulting in closed comedones which are the origin of the subsequent lesions. Effective medications for acne include benzoyl peroxide, topical/systemic retinoids, and topical/systemic antibiotics [6].

Isotretinoin is an oral retinoid and a derivative of vitamin A which has revolutionized the treatment of severe acne in recent years. However, it has significant mucocutaneous and systemic side effects such as teratogenicity and hepatotoxicity [7, 8]. Dryness of lips and nasal mucosa, conjunctivitis, and xerosis are the most common side effects [9–11]. In some cases, these side effects are so severe and annoying for the patient, which results in patient being incompliant and urging the physician to taper or discontinue the medication.

Omega-3 fatty acids (n-3 FAs) are a group of long-chain and very-long-chain polyunsaturated fatty acids (PUFAs) [12]. Since they cannot be produced by human body, their dietary intake is essential [13]. The main sources of omega-3 PUFAs are fish oils [14] and plants [15]. According to the 2012 National Health Interview Survey, omega-3 is a safe supplement without any major side effect. Omega-3 not only does not have any adverse effect on liver, but also can improve serum lipid profile by decreasing serum triglyceride and total cholesterol levels and increasing HDL cholesterol concentration [16]. Furthermore, several clinical trials have confirmed the efficacy of omega-3 in treatment of depression [17]. Lipid mediators derived from omega-3 fatty acids are anti-inflammatory molecules and play a major role in resolution of inflammation [18]. Some studies have reported their effectiveness in resolution of eczematous lesions [19] and prevention of allergic diseases [20]. Kangari et al. observed the effects of omega-3 in alleviating symptoms of dry eye syndrome [21]. In a study by Bhargava and Kumar on patients with contact lens-related dryness of eye, omega-3 significantly reduced this complication [22]. Creuzot et al. also proved the improvement of dry eye symptoms following administration of omega-3 [9]. In a study by Barcelos et al., administration of omega-3 reduced epidermal water loss and thus reduced xerosis symptoms in rats [23].

The impact of oral omega-3 on mucocutaneous side effects of isotretinoin in patients with acne vulgaris has not been evaluated yet. Since omega-3 is an inexpensive and available oral supplement with no adverse effect, if it proved to be effective in controlling mucocutaneous side effects of isotretinoin, it can be prescribed for patients receiving oral isotretinoin, resulting in more satisfaction and compliance of patients.

## 2. Materials and Methods

This case-control study included 118 patients with acne vulgaris, presenting to our dermatology clinic between May 2014 and May 2015. Patients with moderate or severe acne who did not respond to conventional therapy were enrolled after obtaining their written consent. The study was approved by our institutional ethics committee. Patients with hyperlipidemia, hepatitis, seafood allergy, or bleeding disorders; pregnant or lactating women; those who did not intend to use long-term contraception; and those who were receiving anticoagulant drugs, omega-3, or vitamin A derivates were excluded from the study.

Patients were randomly divided into two groups. The case group received oral isotretinoin (0.5 mg/kg) plus omega-3 soft gel capsules (1 g/day; Nature Made Company, CA, USA) and the control group received only oral isotretinoin with the same dose for 16 weeks. Mucocutaneous side effects of isotretinoin, including scaling/dryness of skin, cheilitis, crust formation in the nose, epistaxis, and conjunctivitis, were assessed in both groups by a dermatologist who was blind to the study at baseline and weeks 4, 8, 12, and 16.

Finally, these side effects were compared in two groups using chi-square test and *t*-test. All statistical analyses were performed using SPSS 16.0 (SPSS Inc., Chicago, IL, USA). *P* < 0.05 was considered as statistically significant.

## 3. Results

A total of 104 patients completed the study. Fourteen patients did not agree to complete the study because of remission of their lesions after the first prescription of isotretinoin. The case group included 50 patients and the control group included 54. The subjects were 67 females (64.4%) and 37 males (35.6%). Acne lesions in 77 cases (74%) were limited to the face and in 27 cases (36%) were located on both face and trunk. Eighty patients (77%) had severe (nodulocystic) acne and 24 patients (23%) had moderate (papulopustular) acne. The mean age of patients was 22.8 ± 4.9 years and the mean duration of the disease was 4.9 ± 3.6 years.

The two groups had no significant differences in age and mean duration of the disease. The two groups were also matched for the frequency of sex and location of acne lesions. (Table 1).

Table 2 summarizes mucocutaneous side effects of isotretinoin in case and control groups. Dry lips were significantly less frequent in case group in weeks 4, 8, and 12. Dry nose was found to be less frequent in the group treated with isotretinoin and omega-3 in comparison to the group treated with isotretinoin alone in weeks 4, 8, 12, and 16. Also, the dryness of skin in weeks 4, 8, 12, and 16 was significantly less frequent in the group treated with isotretinoin and omega-3 in comparison to the group treated with isotretinoin alone. Dry eyes were significantly less frequent in case group only in week 4, but it did not show significant difference between two groups in weeks 8, 12, and 16.

## 4. Discussion

In recent years, retinoids have been administered in a wide range of clinical conditions. Besides their several indications and well-established efficacy in dermatology, they are under investigation for their possible role in treatment and prevention of different kinds of malignancies [24, 25]. However, retinoid therapy may be complicated by some side effects, especially mucocutaneous toxicity, which can reduce patient compliance and result in tapering or even discontinuation of the treatment.

Currently, omega-3 PUFAs are in extensive research, particularly for their preventing and treating effects in organ-specific inflammation. They have also been shown to play a protective role in some degenerative diseases such as rheumatoid arthritis [25], type 2 diabetes mellitus [26], autoimmune disorders, cardiovascular diseases [12, 27], and malignancies [28, 29].

There are few studies concerning administration of omega-3 PUFAs to reduce side effects of retinoids [30–32]. The only trial investigating the role of omega-3 on triglyceride levels in patients receiving isotretinoin found that supplementation with omega-3 may be a useful way to manage lipid levels in these patients [32].

In the present study, administration of omega-3 reduced isotretinoin-induced xerosis. This might be attributed to increased skin hydration. A recent study by Barcelos et al. demonstrated that fish oil supplementation reduces the transepidermal water loss, increases the skin hydration, and consequently decreases skin dryness and pruritus in rats [23].

We found that dryness of eyes was less frequent in patients receiving omega-3. There are several additional studies supporting our result. In a recent randomized, double-blind, multicentric trial, Bhargava and Kumar showed benefits of oral omega-3 in alleviating dry eye symptoms [22]. In another study by Kangari et al., oral administration of omega-3 fatty was associated with a decrease of tear evaporation, an increase in tear secretion, and eventually improvement of dry eye symptoms [21]. However, none of these studies were performed in patients receiving isotretinoin.

Furthermore, we demonstrated that cheilitis and dryness of nose mucosa improved by administration of omega-3 in patients who were receiving isotretinoin. To our knowledge, the effects of omega-3 on these complications were not evaluated in any of the previous studies.

## 5. Conclusions

Since oral omega-3 reduces mucocutaneous side effects of isotretinoin, it is recommended in patients with acne vulgaris who are receiving this drug. Future prospective randomized placebo-controlled trials with different doses and formulations of isotretinoin and omega-3 are required to confirm this hypothesis.

## Figures and Tables

**Table 1:** Comparison of the frequency of sex and location of acne in case and control group.

	Isotretinoin group *N* (%)	Isotretinoin + omega-3 group *N* (%)	*P* value
*Sex*			
Male	19 (35)	18 (36)	0.931
Female	35 (65)	32 (64)	
*Acne location*			
Face	37 (68.5)	40 (80)	0.182
Face and trunk	17 (31.5)	10 (20)	

**Table 2:** Comparison of the frequency of mucocutaneous side effects of isotretinoin between case and control groups.

	Isotretinoin group (%)	Isotretinoin + omega-3 group (%)	*P* value
*Dry lips*			
Baseline	0	0	
Week 4	78.7	58	0.030^*∗*^
Week 8	64.8	50	0.041^*∗*^
Week 12	44.4	26	0.044^*∗*^
Week 16	25.6	14	0.130
*Dry nose*			
Baseline	0	0	
Week 4	33.3	12	0.010^*∗*^
Week 8	24.1	10	0.003^*∗*^
Week 12	14	2	0.020^*∗*^
Week 16	11.1	0	0.001^*∗*^
*Dry skin*			
Baseline	0	0	
Week 4	40.7	16	0.003^*∗*^
Week 8	22.2	10	0.021^*∗*^
Week 12	18.5	8	0.002^*∗*^
Week 16	11.1	2	0.013^*∗*^
*Dry eyes*			
Baseline	0	0	
Week 4	13	4	0.046^*∗*^
Week 8	9.3	6	0.533
Week 12	3.7	0	0.169
Week 16	0	0	1.00

^*∗*^ *P* values less than 0.05 were considered significant.
